# MRI features of perifibrinous deposits in the placenta due to COVID-19

**DOI:** 10.1259/bjrcr.20220132

**Published:** 2023-02-07

**Authors:** Hiba Alessa, Luiz Cesar Peres, Emma Ferriman, Andrew Fry, Elspeth Whitby

**Affiliations:** 1 Reproductive and Developmental Medicine, University of Sheffield, Sheffield, United Kingdom; 2 Sheffield Children’s Hospital, Sheffield, United Kingdom; 3 Sheffield Teaching Hospitals NHS Foundation Trust, Sheffield, United Kingdom

## Abstract

COVID-19 has been linked to pregnancy complications and loss (1). Infection during pregnancy is usually mild (2). The risk is highest in the third trimester with increased hospital admission rates and maternal and fetal compromise (3). Post-COVID placentitis is uncommon but the effect on the placenta and the fetus is extensive (4). We present a case correlating clinical, imaging, and pathological findings. **Case Report:** A 29-year-old para 2 gravida 1, with a normal fetal anomaly scan at 22 weeks gestational age (GA) contracted COVID at 24 weeks gestation. Fully recovered but reported reduced fetal movements at 27 weeks and 1 day. **Imaging:** US scan showed bright echoes within the brain, small lungs, and oligohydramnios. MRI showed abnormal brain signals, small lungs, and oligohydramnios but also a very abnormal placenta. Reduced and heterogeneous T2 signal and a marked reduction in the DWI signal intensity. The placental size was markedly reduced (volume 785.6 cm3 expected for GA is 5604.8–5952.4 cm^3^. The surface area of attachment was 3220 mm^2^, expected 22180.4–29293.2 mm^2^). **Pathology:** The placenta was small (fifth centile) with massive perivillous fibrin deposition and multifocal chronic deciduitis. Histology revealed placental chorionic villi showing diffuse sclerotic changes surrounded by perivillous fibrin deposition in the intervillous space. The basal plate revealed multifocal chronic deciduitis. When imaging the fetus, it is important to examine the placenta and correlate any abnormalities. The placenta is a forgotten organ and should be routinely included and assessed to allow the detection of important abnormalities.

## Introduction

COVID-19 has been linked to pregnancy complications and loss.^1^ Infection during pregnancy is usually mild.^2^ The risk is highest in the third trimester with increased hospital admission rates and maternal and fetal compromise.^3^ Although post-COVID placentitis is uncommon, the effect on the placenta and the fetus is extensive.^4^

There is little evidence available describing the placenta *in situ*. Following the COVID-19 pandemic, several researchers reported placental pathology in COVID-infected pregnancies. Their pathological findings were significantly different than those of uninfected placentas but there are no consistent findings. Maternal-fetal disease transmission have also been described. A recent published report of 68 cases from 12 countries described the three most recognisable pathological features which were: chronic histiocytic intervillositis (CHI), increased fibrin deposition (IF), and trophoblast necrosis (TN).^5^ Syncytiotrophoblast cells are the target for the COVID-19 infection as they express ACE2 receptors which influence the viral infection.^5^ There were some cases in which additional cell types were positive for the virus.^5^ In intrauterine fetal death (IUFD), placentas were extensively involved. Only one paper described any MRI findings, and these were obtained following an intrauterine fetal death (IUFD).^6^ Our case reports massive perivillous fibrin deposition (MPFD) and intervillous thrombi previously reported post-COVID-19 infection^4,6^ but is the first report of detection by MRI in an ongoing pregnancy. We present the MRI findings and pathological correlation in the placenta in a case post-COVID-19 infection.

## Clinical presentation

A pregnant woman was reviewed at 22 weeks gestation for abnormal fetal foot position. She was then discharged with reassurance of normality including fetal growth. Two weeks later she tested positive for COVID-19. Two weeks after the COVID-19 infection, she presented with itchy hands and feet, but no abnormality was noticed. Two days later, at 27 weeks and 1 day, she presented with reduced fetal movements. She was referred for a routine growth scan which showed a growth-restricted baby. She had a previous normal vaginal delivery of an appropriately grown baby.

### Investigations and imaging findings

#### Ultrasound scans

A growth scan at 29 weeks and 4 days was performed, revealing a globally small fetus with all measurements below the third centile and oligohydramnios. The umbilical artery pulsatility index (PI) showed absent end-diastolic flow but the middle cerebral artery (MCA), ductus venosus and uterine artery dopplers were normal. The fetal brain images demonstrated a prominent third ventricle containing bright areas. The fetal chest appeared small.

#### Fetal MRI

**Figure 1.**
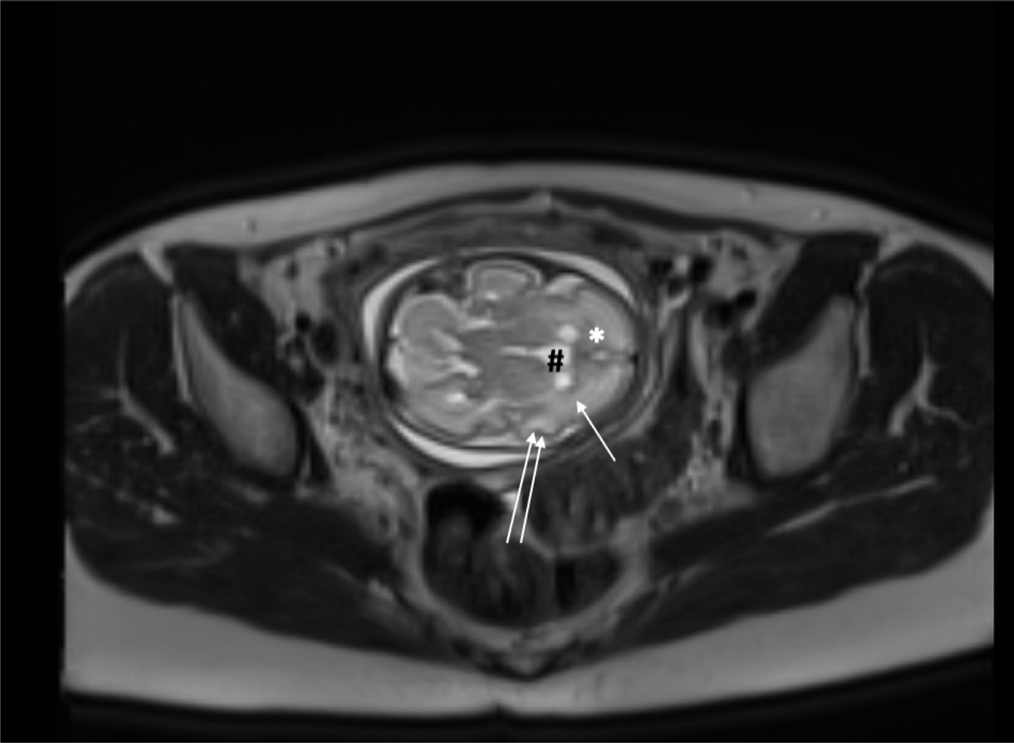
T2 haste axial section fetal MRI, the fetal brain shows abnormal changes (increased differentiation between the white (arrow) and grey matter (double arrow), bulky cavum septum pellucidum (#) and prominent neuronal migration pattern, seen as bands in the white matter of the frontal lobe (*)).

An urgent fetal MRI showed a globally small fetus. There was increased differentiation between the white and grey matter, and abnormal DWI pattern, a bulky cavum septum pellucidum and the neuronal migration pattern was still evident (should no longer be present at 29 weeks GA) (Figure 1).

**Figure 2.**
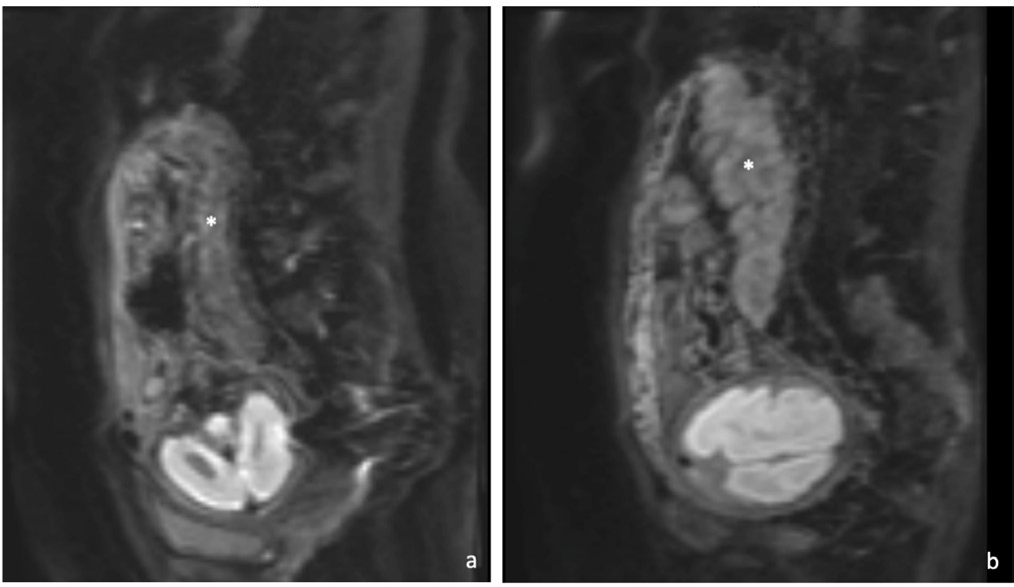
(**a**) Sagittal DWI MRI showing reduced diffusion in the placenta (*), (**b**) DWI MRI of normal fetal MRI scan at 29 weeks gestation, the placenta is uniformly bright (*).

**Figure 3.**
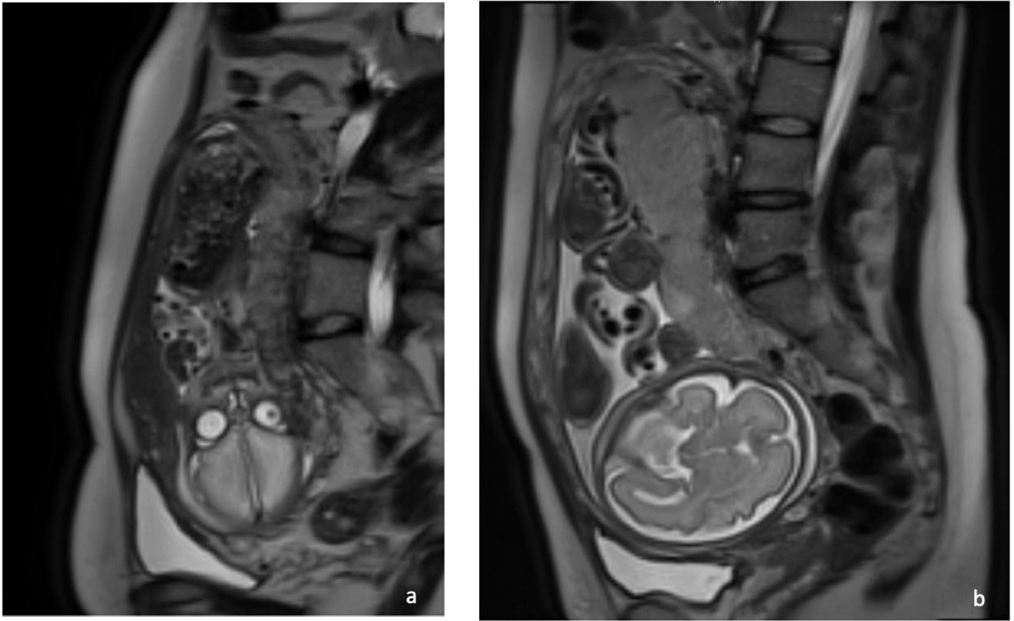
(**a**) *T_2_*-weighted sagittal section MR image demonstrating heterogeneous placental signal and very low signal intensity consistent with the diffuse fibrin deposition. (**b**) *T_2_*-weighted sagittal section MR images of a normal placenta at same gestation.

**Figure 4.**
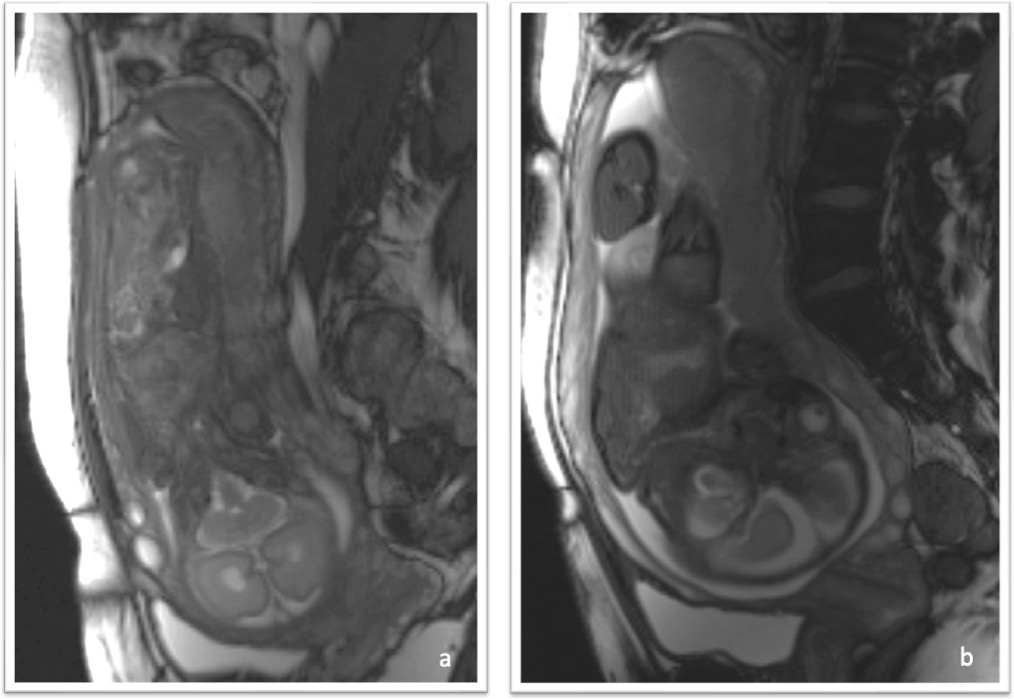
(**a**). Sagittal section of balanced grading echo of COVID-19 placentatits at 29 weeks gestation. (**b**) Sagittal section of balanced grading echo of normal placenta at same gestation.

**Figure 5.**
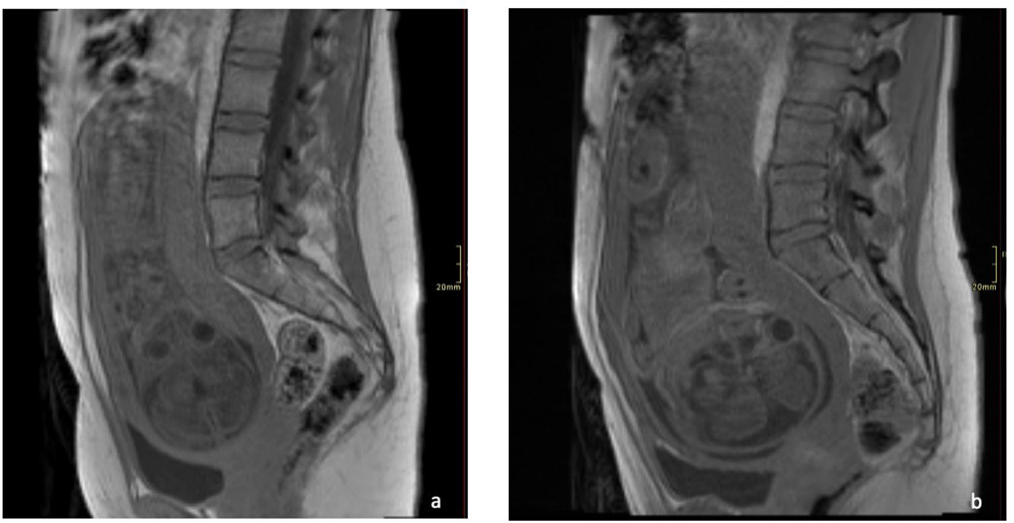
(**a**) T1 fl2d sagittal section of COVID-19 placentitis at 29 weeks gestation. (**b**) T1 fl2d of normal placenta at same gestation.

The placenta was seen to be abnormal. There was very little signal on the diffusion-weighted images (Figure 2a) which should be uniformly bright at this gestational age (Figure 2b). On the T2- weighted images the placenta was of a lower signal intensity than expected and heterogenous (Figure 3a) which was consistent with diffuse fibrin deposition. The Balanced gradient echo (Figure 4a and b) and T1 (Figure 5a and b) sequence signal intensities were not significantly different to that expected at this gestational age.

#### Management and outcome

Cardiotocography was performed which showed reduced variability and reduced short-term variation (STV) of 3.9. The couple were counseled that although there was no definitive diagnosis at this stage there was a high risk of an abnormal outcome.

A watch and wait policy was offered, with a referral for a fetal echocardiogram, a repeat MRI two weeks later and a twice-weekly assessment of liquor and dopplers. The alternative of late termination of pregnancy with a feticide procedure and induction of labor was also given as an option. The couple opted for termination of pregnancy and a feticide procedure was performed. A stillborn male baby was delivered at 30 weeks and 2 days weighing 780 g (expected for 30 weeks is: 1115 ± 329 g).

A post-mortem examination was performed which confirmed growth restriction and showed placental fibrin deposition. All other postnatal investigations were normal including viral infection and thrombophilia screen.

The cause of intrauterine growth restriction was placentitis resulting in massive peri-villous fibrin deposition and chronic deciduitis, as confirmed by the histopathology report. COVID placentitis is the likely explanation since massive perivillous fibrin deposition is a well-recognised lesion in placentas affected with COVID, and in this case immunostaining for SARS-CoV2 was positive in the villous trophoblast.^5^ Investigations excluded coagulation disorders and cytomegalovirus.

#### Postmortem and pathology report

**Figure 6.**
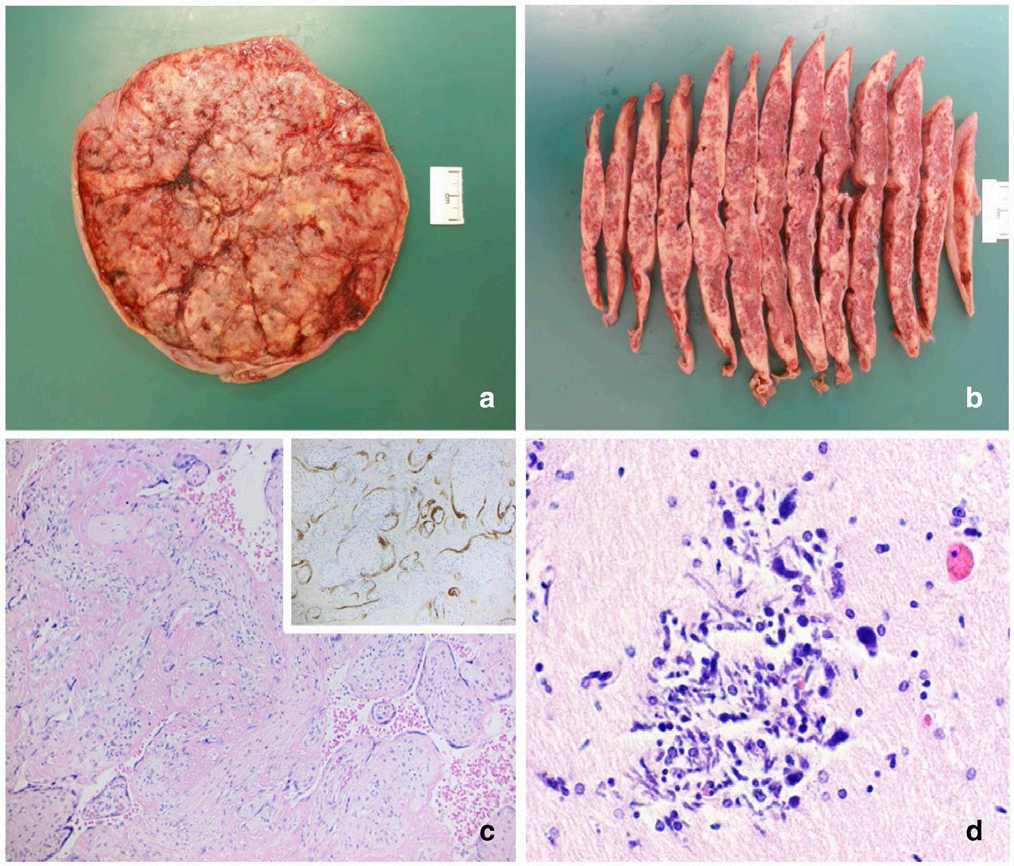
Macroscopic view of the maternal surface of the fixed placental sample showing widespread yellowish material (**A**), which on cut surface is represented by massive perivillous fibrin deposition, imparting a marbling effect (**B**). Histological examination of the placenta stained with haematoxylin and eosin confirmed the massive perivillous fibrin deposition, seen as a diffuse pinkish material surrounding chorionic villi and in the inset is the immunostaining for SARS-CoV2 showing positivity in the syncytiotrophoblast as a brown granular material (C, original magnification x10). The brain section shows a focus of calcified neurones, which represent longstanding hypoxic-ischaemic encephalopathy (D, original magnification x40)

A comprehensive post-mortem examination was performed. The results of the placenta and fetal examination revealed mildly macerated male fetus with no dysmorphic features with weights and measurements consistent with 28 weeks and an atrophic thymus. The placenta was small for the given gestational age (fifth centile) and consisted of massive perivillous fibrin deposition on the cut surface (Figure 6a and b). Histological examination confirmed the widespread perivillous fibrin deposition and immunostaining for SARS-COV2 was strongly positive in the syncytiotrophoblast (Figure 6c). An additional finding was numerous neurons with eosinophilic cytoplasm in the cerebellum and brainstem, indicating hypoxic changes, groups of calcified neurons in the periventricular area, which represent dead cells due to a previous hypoxic insult, as well as fresh and old subarachnoidal haemorrhage. Fetal demise was due to hypoxic-ischaemic changes induced by the abnormalities in the placenta.

#### MR images analysis results

Compared to normal reference values, placenta volume and surface attachment were smaller for the same gestation: Volume 786 cm^3^ (normal 5605–5952), surface area of attachment 3320 mm^2^ (normal 22180–29293 mm^2^). Visually the placenta was very abnormal.

## Discussion

In this report, we have described a third trimester COVID-19 infection associated with placental insufficiency and intrauterine growth restriction. Antenatal MRI findings corresponded with the pathology results. The clinical background and histopathology results were consistent with COVID placentitis, confirmed with immunostaining for SARS-COV2.

The MRI findings were different to those in the one reported case in the literature.^6^ The *T_2_* weighted images were similar with increased heterogeneity and the increased dark areas most likely corresponding to the fibrin deposits. Diffuse heterogeneity is also seen in cases of placenta accreta spectrum, again associated with vascular changes and fibrin deposition but these cases have additional diagnostic findings alongside the heterogeneity that are not seen in this case.

Here the DWI was unusually low whilst in the previous report it was higher than normal. In that case the fetus had already demised. In addition, the previous case was 7 days post infection and our case over 4 weeks post infection and the difference may be explained by the time difference, acute changes associated with thrombosis and more chronic changes due to the thrombosis leading to fibrin deposition.

Perivillous fibrin deposition is a rare entity, that accounts for (0.028%–0.5%) of deliveries, the cause of which is still unknown, but mechanisms linked to maternal autoimmune and alloimmune have been suggested.^7^ Increase fibrin deposition is the most common finding in post COVID placentitis, although It is normal for placentas to contain fibrin deposits to a certain extent. Placental perfusion is compromised when fibrin is superimposed on a damaged placenta. The most severe abnormalities are MPFD and maternal floor infarction.

The degree of placental damage in COVID placentitis is unrelated to the clinical features or severity of infection.^5^ In a clinical statement published by the Royal College of Physicians of Ireland (RCPI), it was recommended that pregnancies complicated by COVID infection be assessed thoroughly when they present with reduced fetal movement.^8^ This followed a report of 6 stillborn and neonatal death cases from January to March 2022, all of which were caused by the new SARS-COV-2 α variant (B.1.1.7) related to most stillbirth cases caused by COVID placentitis.^8^ In November 2021 the US centers for disease control and prevention confirmed most cases of stillbirth were associated with SARS-Cov-2 δ variant (B.1.617.2).^9^ The virus variant was not tested in this case; however the case was diagnosed during the time period when δ was the dominant circulating strain.

Autopsies of 63% of cases revealed no fetal abnormalities. Intrauterine hypoxia was identified as the most common pathologic finding.^5^ The key feature of post-mortem findings with COVID-19 is acute fetal hypoxia where maternal-fetal transmission of infection is uncommon. If damage does happen, it is usually confined to the placenta.^5^ In most cases, doppler ultrasound has no benefit in the assessment of COVID placentitis. For that reason, MRI is recommended in the assessment of COVID placentitis.^5^

In this report, IUGR is most likely the result of placental damage and insufficient transfusion of oxygen and nutrient. Although IUGR features need some time to develop, accumulation of fibrin in the intervillous space could eventually obstruct placental blood flow, resulting in placental insufficiency and stillbirth. The imaging and pathological features are those of COVID19 placentitis in the context of the clinical history and immunostaining. Placental infection through vertical transmission is rare as the virus requires specific receptors, i.e., angiotensin-converting enzyme 2 (ACE2) to attach to, which are not abundant in placenta trophoblastic cells.^10^

## Conclusion

Although it is uncommon, poor fetal outcome may be associated with post COVID placentitis. MRI appearances, including the DWI signal, volume, and surface attachment should be assessed in any pregnancy post COVID-19 where there are clinical concerns for the health of the fetus. We believe this is the first reported case of MRI detecting COVID-19 placentitis in the antenatal period.

## Learning points

- Post COVID-19 placentitis is a rare entity but the severity of placental damage is unrelated to the severity of infection.
- MRI changes showed a heterogenous placenta with lower signal intensity of DWI and T2- weighted images.
- In our case, fibrin deposition and chronic deciduitis caused placental insufficiency, UGR and end organ damage which is consistent with other cases reported.
